# Sudden Cardiac Arrest in a Young Adult: A Diagnostic Challenge

**DOI:** 10.7759/cureus.84481

**Published:** 2025-05-20

**Authors:** Yasser Hegazy, Allison Foster, Md Ripon Ahammed, Ali Assaker, Omar Abdelhalim, Nuha Al-Howthi, Muhammad Ghallab

**Affiliations:** 1 Internal Medicine, Icahn School of Medicine at Mount Sinai, Queens Hospital Center, New York, USA; 2 Internal Medicine, Icahn School of Medicine at Mount Sinai, New York City Health + Hospitals-Queens, New York, USA

**Keywords:** hypertrophic cardiomyopathy (hcm), implantable cardioverter defibrillator, sudden cardiac arrest, ventricular fibrillation, young adult

## Abstract

Sudden cardiac arrest (SCA) is a leading cause of mortality in young individuals, often linked to structural heart disease or primary electrical disorders. However, in some cases, the etiology remains unidentified despite extensive diagnostic efforts. This case report describes a 23-year-old male with a family history of hypertrophic cardiomyopathy (HCM) who experienced a sudden cardiac arrest without prior symptoms and had negative genetic testing. The patient, previously healthy, suffered a cardiac arrest while traveling to college. Advanced cardiopulmonary resuscitation (CPR) and multiple defibrillator shocks were necessary to achieve return of spontaneous circulation (ROSC). Transthoracic echocardiography performed immediately post-ROSC showed global hypokinesia with reduced ejection fraction (35%). Coronary angiography at 24 hours post-ROSC was normal. Transient ST-segment elevations resolved within hours and were attributed to post-resuscitation myocardial stunning, with no evidence of ischemia or myocarditis on cardiac magnetic resonance imaging (MRI), which revealed mild interventricular septal hypertrophy without left ventricular outflow tract obstruction. Genetic testing, including a targeted cardiomyopathy panel and whole exome sequencing, did not identify any pathogenic variants, including in MYH7 or MYBPC3. The patient was treated with amiodarone and received an implantable cardioverter-defibrillator (ICD) for secondary prevention. He recovered fully, with no neurologic deficits. This case underscores the challenges of diagnosing and managing SCA in young adults, emphasizing the critical role of genetic and structural assessments. Early intervention, multidisciplinary care, and comprehensive follow-up are essential to reduce recurrence and improve patient outcomes.

## Introduction

Sudden cardiac arrest (SCA) is a leading cause of mortality worldwide, affecting approximately 300,000 to 400,000 individuals annually in the United States, including 5,000 to 7,000 cases among children and young adults [1]. SCA refers to the abrupt loss of cardiac function and can be reversible with immediate resuscitation. In contrast, sudden cardiac death (SCD) is the fatal outcome of SCA when return of spontaneous circulation (ROSC) is not achieved. SCA in younger populations poses unique diagnostic and management challenges, with etiologies ranging from structural heart diseases such as hypertrophic cardiomyopathy (HCM) to primary electrical disorders like long QT syndrome (LQTS) and Brugada syndrome (BrS) [2,3]. Notably, up to 30% of SCD in individuals under 35 years old remain unexplained even after comprehensive evaluation, often labeled as sudden arrhythmic death syndrome (SADS) [4]. Genetic factors play a significant role in many cases, and the advent of next-generation sequencing has improved the identification of hereditary conditions, although limitations such as incomplete penetrance and complex gene interactions persist [3,5]. This case report highlights a 23-year-old male who suffered an out-of-hospital SCA with ventricular fibrillation (VF) as the presenting rhythm. Despite a significant family history of HCM, comprehensive evaluation, including imaging, genetic testing, and endomyocardial biopsy, failed to identify a definitive cause. Coronary anomalies, including anomalous origin of coronary arteries, were specifically excluded via coronary angiography. The case underscores the complexity of diagnosing SCA in young adults with normal coronary arteries and negative genetic findings. It emphasizes the critical importance of early intervention, including defibrillator placement, and the need for further research to elucidate the underlying mechanisms of unexplained SCA in this population.

## Case presentation

A 23-year-old male with a past medical history of asthma, seasonal allergies brought into the hospital by emergency medical service (EMS) after sustaining a sudden cardiac arrest while he was on his way to his college. The patient’s mother was driving him to the college. The patient suddenly spilled his drink followed by becoming unresponsive. His mother reported that the patient did not complain of anything before the event and they were laughing & playing around, then all of a sudden, he stopped talking, spilled his drink (mild Lipton tea), lost his breath and became unresponsive. She immediately called the EMS, and the patient was found to be apneic, pulseless with non-recordable blood pressure, cyanotic, without any signs of trauma. According to EMS, ventricular fibrillation was found on the monitor and first CPR was started approximately seven minutes after the estimated cardiac arrest, with administration of three direct current (DC) shocks but no medication. Return of spontaneous circulation (ROSC) was achieved in approximately 20 minutes during transport to the emergency department (ED). Upon arrival in the ED, the patient was again found to be in agonal breathing, with ventricular fibrillation on monitor and ROSC was obtained following intubation with mechanical ventilation and administering two more shocks, one shot of epinephrine, and one shot of bicarbonate. The patient was started on amiodarone for rhythm control.

Of note, at the third month of the patient's intrauterine life, his father died at the age of 38 years due to a sudden cardiac death with autopsy findings consistent with possible hypertrophic cardiomyopathy (HCM). Also, his paternal uncle was diagnosed as HCM at the age of 30 and alive. The patient was followed by a cardiologist periodically since birth and had normal cardiac evaluations. However, the patient was referred to a tertiary-level center for further evaluation, but didn’t undergo genetic testing yet. The patient had asthma during childhood and had not had exacerbations for a long time and didn’t require any inhalers or other asthma medications for years. Patient’s history was negative for any shortness of breath, chest pain, palpitation, syncope, dizziness, lightheadedness on exertion and/or at rest.

**Figure 1:**
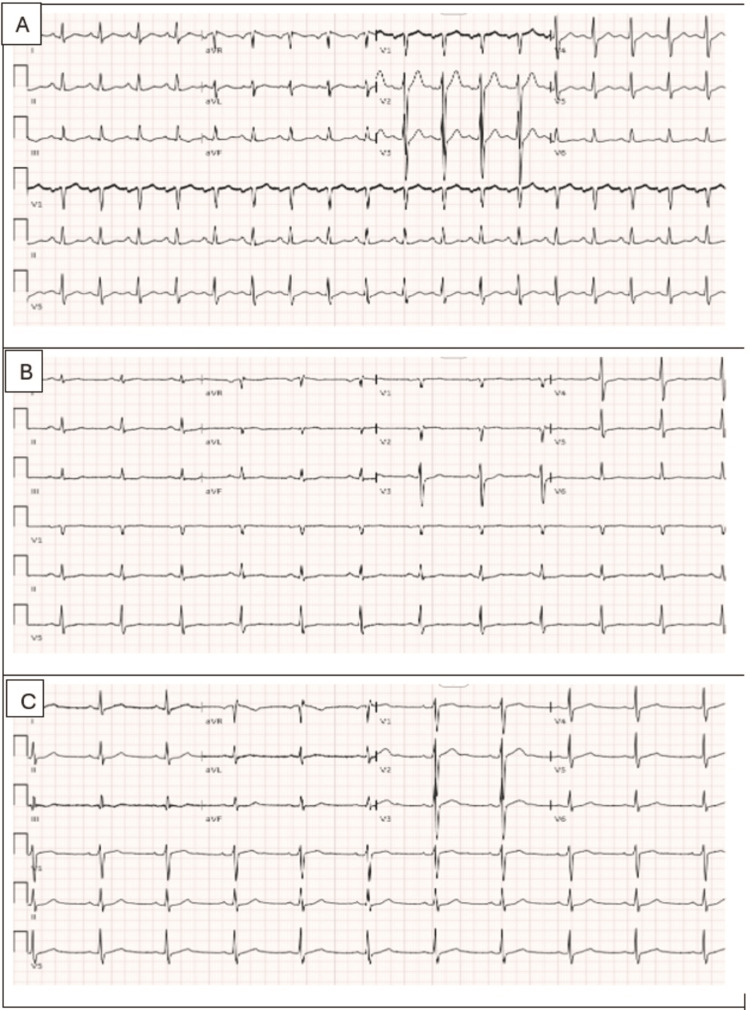
(A) Initial ECG 1: Sinus rhythm with non-specific 0.5 mm down-sloping ST depression in lead III. (B) Second ECG after six hours: sinus rhythm, 1 mm ST elevations in aVR and aVL, 1.5-2 mm horizontal ST depressions in lead III and aVF, 1 mm slow up sloping ST depressions in V5-V6, 2 mm up sloping ST depressions in V3-V4. (C) Third ECG after normal coronary angiogram: normal ECG findings. ECG: electrocardiogram; aVL: augmented vector left; aVR: augmented vector right; aVF: augmented vector foot.

Initial laboratory evaluation post-ROSC showed acute kidney injury, mixed respiratory and metabolic acidosis, and elevated liver enzymes, consistent with global hypoperfusion. Electrolytes and complete blood count were within normal limits. Coagulation parameters were unremarkable. Initial cardiac enzymes were normal, but repeat troponin levels drawn six hours later trended up to 8.750 ng/mL (Table 1). An electrocardiogram (ECG) revealed transient ST elevation in aVR with reciprocal depressions in inferior, anterior, and lateral leads, suggesting global myocardial ischemia without a specific infarction pattern (Figure 1). Head CT ruled out intracranial hemorrhage or anoxic injury, and chest CT was negative for pulmonary embolism. Urine drug screen was negative.

**Table 1:** Initial laboratory findings. ALT: alanine aminotransferase; AST: aspartate aminotransferase; INR: international normalized ratio.

Parameter	Result	Reference range
pH	7.00	7.35-7.45
PCO₂	90 mmHg	35-45 mmHg
Lactate	11.6 mmol/L	0.5-2.2 mmol/L
HCO₃ (bicarbonate)	21 mmol/L	22-28 mmol/L
Serum creatinine	1.30 mg/dL	0.6-1.2 mg/dL
AST	110 U/L	10-40 U/L
ALT	61 U/L	7-56 U/L
Sodium	143 mmol/L	135-145 mmol/L
Potassium	3.5 mmol/L	3.5-5.1 mmol/L
Hemoglobin (Hgb)	16.0 g/dL	13.5-17.5 g/dL
White blood cell (WBC) count	10,730 /mcL	4,000-11,000 /mcL
Platelet count	163,000 /mcL	150,000-450,000 /mcL
Prothrombin time (PT)	11.5 sec	10-13 sec
INR	1.0	0.8-1.2
Initial troponin T	<0.010 ng/mL	<0.01 ng/mL
Repeat troponin in six hours	8.750 ng/mL	<0.01 ng/mL

**Figure 2:**
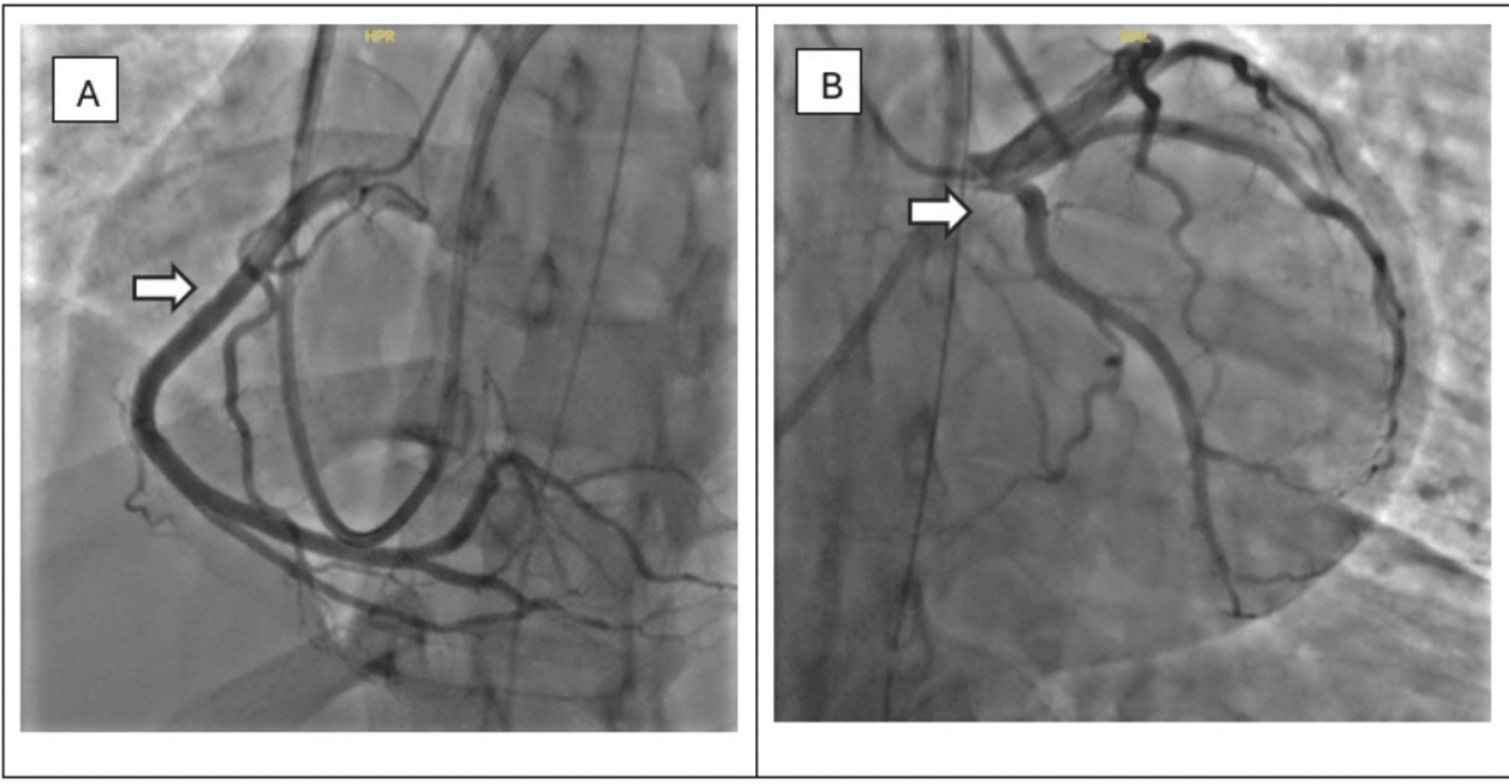
(A) Arrow indicates the normal right coronary artery and (B) arrow indicates the normal left main coronary, left anterior descending, and left circumflex arteries.

**Figure 3:**
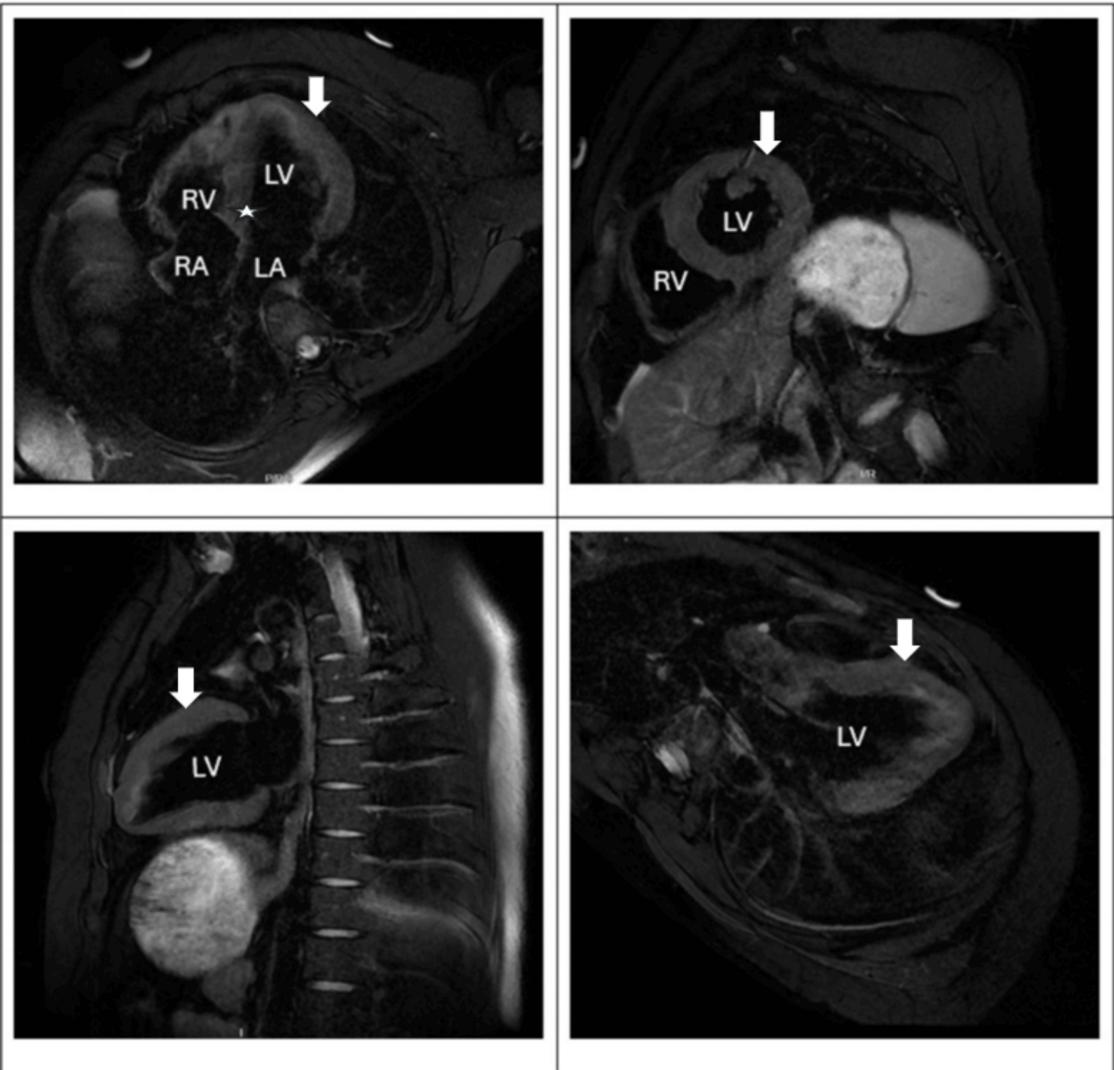
Cardiac MRI with and without contrast. Arrows indicate normal left ventricular size with normal systolic function; no abnormal late gadolinium enhancement. Asterisk indicates mild hypertrophy of the interventricular septum measuring up to 15 mm in the basal anterior septum. No evidence of left ventricular outflow tract obstruction. MRI: magnetic resonance imaging; LA: left atrium, LV: left ventricle, RA: right atrium, RV: right ventricle.

The patient was transferred to the medical intensive care unit (MICU) for further management. He was managed with targeted temperature management and started on amiodarone for rhythm control. Transthoracic echocardiography (TTE) revealed biventricular hypokinesia with a reduced left ventricular ejection fraction (LVEF) of 35%. Cardiology recommended transferring the patient to a tertiary-level facility for further workup, including a coronary angiogram. Workup in a tertiary hospital revealed a normal coronary angiogram (Figure 2). Subsequently, both TTE and transesophageal echocardiogram (TEE) were normal with normal ejection fraction. ECG normalized too with trending down of troponin. The hospital course was complicated by aspiration pneumonia, which was treated successfully with vancomycin and piperacillin/tazobactam. The patient’s condition started to improve, as evidenced by the improvement in mental status and normalization of the above-mentioned abnormal labs. Magnetic resonance imaging (MRI) of heart with and without contrast revealed a normal left ventricle in size and shape, with left ventricular ejection fraction 52%; no abnormal late gadolinium enhancement; mild hypertrophy of the interventricular septum measuring up to 15 mm in the basal anterior septum (Figure 3). No evidence of left ventricular outflow obstruction. Endomyocardial biopsy revealed mild hypertrophy and patchy interstitial fibrosis in trichrome stain; no evidence of myocarditis was seen; Congo red stain and iron special stain were negative. These findings, in conjunction with MRI, were considered potentially consistent with an early or atypical HCM phenotype. Genetic testing was negative.

Comprehensive genetic testing, including a cardiomyopathy panel, was negative for known pathogenic variants. Infectious and autoimmune workup, including viral serologies, bacterial screening, and extended rheumatologic panels, was unremarkable. A weakly positive antinuclear antibody (ANA) (speckled pattern, low titer) was considered incidental, with no additional clinical or serologic evidence of autoimmune myocarditis. An electrophysiology (EP) study was performed to assess for occult conduction abnormalities or inducible arrhythmias. No pre-excitation or inducible tachyarrhythmias were found. Despite this, an implantable cardioverter-defibrillator (ICD) was placed for secondary prevention given the VF arrest. The patient was weaned off amiodarone during recovery, as no further arrhythmias were noted. His hospital course was complicated by aspiration pneumonia, which was treated successfully. He was discharged hemodynamically stable, at baseline mental status, with only mild procedural site pain, and was scheduled for outpatient cardiology follow-up. Device interrogation at discharge showed no arrhythmic events.

## Discussion

Sudden death is defined as an unexpected fatal event within an hour of symptom onset or within 24 hours of last seen well, not due to homicidal, suicidal cause, or an accident. Nearly 70% of the causes of sudden death are due to sudden cardiac death (SCD), with an estimated number of 300,000-400,000 affected people yearly in the United States; 5000-7000 of them are children [6]. In other words, SCD accounts for around 20% of deaths in Western countries [7]. The incidence has declined recently due to recent advances in prevention and management, especially with the growing use of implantable cardiac defibrillators [8]. The causes of SCD vary according to the different age cohorts, with coronary artery disease (CAD) and cardiomyopathy constituting around 40% of the causes in people under 35 years old and almost 85% of the causes in those above 35 years old [6]. In the former group, after forensic analysis, the cause of SCD is non-identifiable in 30% of the cases [7]. Other causes of SCD in this age group include structural heart diseases and primary electrical disorders. Environmental and genetic causes have been implicated in SCD, with the latter has been widely tested through next-generation sequencing [6,8].

When it comes to the structural etiologies, coronary artery disease (CAD) constitutes 50% of the causes of SCD. In the Oregon SCD study, CAD was the second most common cause of SCD in individuals between five and 34 years old. Although the risk is highest in the first month after myocardial infarction, almost two-thirds of CAD-related SCDs occur in supposedly low-risk subjects. The cause of CAD varies with age, with atherosclerosis being the culprit for older individuals. Anomalous coronary arteries are specifically seen in athletes; although left main arising from the pulmonary artery is rarely seen beyond infancy, a malignant course between the aorta and the pulmonary artery can be seen in older individuals. Non-ischemic cardiomyopathies are responsible for up to 30% of the causes of SCD in the young cohort, and they include hypertrophic, restrictive, and dilated cardiomyopathies. Other causes include arrhythmogenic right ventricular cardiomyopathy, myocarditis, Marfan Syndrome, and left ventricular non-compaction [6,8]. Valvular heart disease is responsible for almost 1-5% of SCDs in native valves and up to 1% in prosthetic valves. This is higher among subjects with severe aortic stenosis or mitral valve prolapse. The pathophysiology could be either mechanical or arrhythmogenic [9]. Hypertrophic cardiomyopathy is the most common inherited cardiovascular disorder linked to SCD in the young, often presenting with little to no warning signs, as in our patient. Familial cases, especially with a history of early sudden death, significantly increase risk.

In the absence of structural heart abnormalities, primary electrical disorders are frequently implicated in sudden cardiac death (SCD). These include long QT syndrome (LQTS), Brugada syndrome (BrS), catecholaminergic polymorphic ventricular tachycardia (CPVT), short QT syndrome, and Wolff-Parkinson-White syndrome [6,8]. Advances in genomic research have identified approximately 49 genes associated with these arrhythmogenic conditions [8]. Next-generation sequencing has emerged as a valuable tool for detecting pathogenic variants associated with these syndromes, facilitating the identification of individuals at increased risk for SCD [6]. For instance, targeted sequencing of genes implicated in LQTS, BrS, and CPVT can reveal disease-causing mutations. Nevertheless, genetic testing remains limited by factors, such as incomplete penetrance, polygenic inheritance patterns, and gaps in current genomic knowledge that may hinder result interpretation [8]. In the present case, despite a strong clinical suspicion based on family history, genetic testing yielded negative results--highlighting these limitations. Therefore, comprehensive clinical assessment and family-based cascade screening remain essential components of risk stratification, even when genetic findings are inconclusive.

The pathophysiology of SCD varies based on the initial cause, with ventricular fibrillation is the culprit in acute coronary ischemia, while ventricular tachycardia is the cause in cases of structural heart diseases and channelopathies. Another potential cause of SCD is catecholamine surge, which could lead to calcium handling abnormalities within the myocytes and subsequent ventricular arrhythmias. Other possibilities include metabolic derangements and reactive oxygen species production during periods of stress, such as myocardial ischemia. Loss of interaction between heart, lung, brain, and vasculature plays a major role in SCD; when a function of one of these vital organs deteriorates, cessation of function of the other organs follows [10]. Recently, the most common encounter for SCD is asystole or pulseless electrical activity, which is a future challenge [11]. In our patient, the presence of mild septal hypertrophy and interstitial fibrosis on biopsy raises the possibility of an early or atypical HCM phenotype, which may have predisposed him to malignant arrhythmia even in the absence of overt hypertrophy or obstruction.

Given that the overall survival rate following sudden cardiac death (SCD) remains approximately 10%, emphasis is placed primarily on prevention. Preventive strategies are guided by the underlying etiology. Pharmacologic therapy plays a critical role; guideline-directed medical therapy is recommended in patients with myocardial infarction and heart failure to reduce the risk of SCD. Inherited channelopathies such as long QT syndrome (LQTS), Brugada syndrome (BrS), and catecholaminergic polymorphic ventricular tachycardia (CPVT) may be managed with agents including quinidine, flecainide, and mexiletine. Beta-blockers are considered first-line therapy in these conditions, with amiodarone used in select cases. Combination antiarrhythmic therapy may be employed when monotherapy proves inadequate.

Implantable cardioverter-defibrillators (ICDs) remain the cornerstone of SCD prevention and are indicated for both primary and secondary prevention. In this case, the patient underwent ICD implantation for secondary prevention following a documented episode of ventricular fibrillation. ICDs are associated with improved long-term outcomes in survivors of malignant arrhythmias. Catheter ablation may be considered in cases of recurrent ventricular tachycardia, particularly when antiarrhythmic therapy fails or is contraindicated. However, repeat procedures may be required in approximately 15% of patients with structural heart disease and 22% of those without. Other acute interventions include defibrillation at the time of arrest, revascularization in selected patients with ischemic substrate, and targeted temperature management in comatose survivors of SCD [5]. Additionally, lifestyle modifications and trigger avoidance are essential components of prevention. For instance, patients with hypertrophic cardiomyopathy (HCM) are advised to avoid competitive athletics; those with LQTS type 1 should avoid exertional activities such as swimming, and patients with LQTS type 2 should minimize exposure to sudden auditory stimuli or emotional stress when feasible [12].

Finally, as a substantial cause of SCD in the young cohort has a genetic basis, investigating the surviving family members could help in preventing SCD in them. These investigations are tailored to the history, examination, and pertinent investigations. As most of the SCD-related genetic heart diseases are autosomal dominant, genetic testing is emerging as a crucial tool in the early detection of affected family members and in applying preventive measures when possible. For example, genetic screening for LQTS and HCM is now widely available. With that being said, for every genetic cardiac disease, there is a proportion of patients who do not have a gene mutation in the known causative gene or who may have more complex genetic diseases, which is challenging [12].

## Conclusions

This case underscores the diagnostic challenges in evaluating young individuals presenting with sudden cardiac arrest, particularly when initial electrocardiographic and imaging studies are unremarkable. Despite comprehensive testing, including cardiac biopsy and genetic analysis, no definitive etiology was identified--highlighting the limitations of current diagnostic tools and the need for clinical vigilance. The presence of a significant family history of sudden cardiac death, even in the setting of negative genetic testing, reinforces the critical role of thorough family screening and clinical risk stratification. Implantable cardioverter-defibrillator (ICD) placement for secondary prevention proved lifesaving in this case and remains a cornerstone of management in survivors of ventricular fibrillation. Moving forward, research into advanced genetic mapping, improved phenotypic risk models, and the development of accessible screening tools is essential. Public health education should also target families with a history of unexplained cardiac deaths to promote early recognition and timely evaluation. Ultimately, this case emphasizes the importance of a multidisciplinary and proactive approach in managing young patients at risk for sudden cardiac death, even when standard diagnostics are inconclusive.
